# Transfer of trastuzumab and pertuzumab into human breast milk: a case report

**DOI:** 10.1007/s00280-026-04901-0

**Published:** 2026-05-20

**Authors:** Pauline L. M. Buitelaar, Dick Pluim, Sandy Hanssens, Yohan Kerbage, Hilde Rosing, Alwin D. R. Huitema, Frédéric C. Amant

**Affiliations:** 1 Department of Pharmacy & Pharmacology, Netherlands Cancer Institute – Antoni van Leeuwenhoek Hospital, Amsterdam, The Netherlands; 2 Department of Gynaecology and Obstretics, Lille University Hospital (CHU), Lille, France; 3 Department of Gynaecologic Surgery, University Hospital Lille (CHU), Lille, France; 4 OncoThAI: Laser Assisted Therapies and Immunotherapies for Oncology, University of Lille, Lille, France; 5 Department of Clinical Pharmacy, University Medical Center Utrecht, Utrecht University, Utrecht, The Netherlands; 6 Princess Máxima Center for Pediatric Oncology, Utrecht, The Netherlands; 7 Department of Gynaecologic Oncology, University Hospital (UZ) Leuven, Leuven, Belgium; 8 Department of Oncology, KU Leuven and Leuven Cancer Institute, Leuven, Belgium; 9 Department of Gynecology, Netherlands Cancer Institute – Antoni van Leeuwenhoek Hospital, Amsterdam, the Netherlands

**Keywords:** Pertuzumab, Trastuzumab, Breast milk, Breast cancer, Pharmacokinetics

## Abstract

**Purpose:**

Lactation could offer benefit to the mother and child. Information on the safety of lactation during anti-cancer treatment, however, is scarce. Therefore, clinical data about drug exposure through breast milk is needed. To contribute to this, we present a case of a 34-year old woman who was treated with intravenous trastuzumab 600 mg and pertuzumab 1200 mg in the first cycle. Over the first 17 days of treatment, she collected breast milk samples.

**Methods:**

Trastuzumab and pertuzumab concentrations in breast milk were determined by validated ELISAs. Subsequently, infant exposure parameters such as infant daily dose (IDD), relative infant dose (RID), and cumulative RID were calculated.

**Results:**

The cumulative RID was 16.8% for trastuzumab and 24.3% for pertuzumab. Based on our results, we can conclude that intravenously administered trastuzumab and pertuzumab transfer into breast milk exceeding the safety threshold of an RID of 10%.

**Conclusion:**

Although the extent of systemic exposure in infants after ingestion of breast milk containing these monoclonal antibodies is unknown, systemic exposure cannot be excluded. Therefore, breastfeeding during treatment with trastuzumab and pertuzumab should be discouraged.

**Supplementary Information:**

The online version contains supplementary material available at 10.1007/s00280-026-04901-0.

## Introduction

Breast cancer is diagnosed in approximately one in 3000 pregnancies every year, most commonly in women aged 32 to 38 years [1]. Anti-cancer treatment during pregnancy and lactation, however, could be associated with potential developmental risks to the fetus and newborn. Consequently, therapeutic decision-making must balance maternal benefit against potential harm to the child [2]. Because clinical trials in pregnant and breastfeeding women are unethical, evidence-based guidelines for treating these populations are limited, and treatment recommendations depend mostly on published case reports, clinical experience, and theoretical considerations. Safety data for IgG_1_ monoclonal antibodies trastuzumab and pertuzumab, used for treatment of positive metastatic breast cancer, is also lacking. Based on theoretical considerations, LactMed^®^ states that transfer of trastuzumab and pertuzumab into breast milk is likely to be very low and that oral absorption by the infant is expected to be minimal due to degradation in the gastrointestinal tract [3, 4]. Despite this rationale, the absence of clinical data has led manufacturers to advise against breastfeeding during treatment, especially in the neonatal period. Two other therapeutic monoclonal antibodies, nivolumab and infliximab, have been shown to transfer into human breast milk [5, 6], with the neonatal fragment crystallizable receptor (FcRn) potentially being involved [7]. For infliximab, this even resulted in systemic infliximab levels in the breastfed infant, further supporting the possibility of monoclonal antibody exposure in a newborn via breastfeeding [6]. Given that lactation offers substantial benefits for both mother and infant, such as reduced risks of diarrhea and common childhood illnesses in the short term and a potential reduced risk of overweight and obesity later in life [8], more data is needed to guide breastfeeding recommendations during treatment with monoclonal antibodies.

In this case report, we aimed to address this knowledge gap by determining trastuzumab and pertuzumab concentrations in human breast milk collected over the first 17 days following the first treatment cycle.

## Case description

A 34-year-old woman was diagnosed with metastatic breast cancer (invasive lobular carcinoma) during pregnancy. Treatment was initiated during pregnancy, and she received five cycles of 75 mg docetaxel. After four cycles, she achieved a near-complete response, and the remainder of her treatment plan was subsequently defined. As soon as clinically feasible postpartum, she was scheduled to start trastuzumab 600 mg and pertuzumab 1200 mg intravenously (IV) in the first cycle, followed by trastuzumab 600 mg and pertuzumab 600 mg from the second cycle onward. Trastuzumab and pertuzumab treatment was combined with tamoxifen 20 mg daily. Breast milk was collected until lactation ceased, resulting in 17 days of samples obtained during the first cycle of trastuzumab and pertuzumab therapy. Although the patient proactively offered to donate breast milk for research, written informed consent was obtained before the start of breast milk collection.

## Materials and methods

### Drug level measurements

ELISA was selected for the measurement of the possibly very low trastuzumab and pertuzumab concentrations in human breast milk. For trastuzumab, we partially validated an anti-trastuzumab sandwich-ELISA (Trastuzumab (Herceptin^®^) BioAssay™ ELISA Kit by US Biologics) for suitability with human breast milk matrix instead of serum. For pertuzumab, we developed and partially validated an in-house anti-pertuzumab sandwich ELISA. Detailed descriptions of the evaluation of these ELISAs for both drugs are provided in Supplementary Materials S1 and S2.

### Infant drug exposure calculations

The relative infant dose (RID) represents the proportion of a maternal drug dose to which an infant is exposed on a given day. By summing the RIDs across multiple days, the infant’s total exposure over a treatment cycle can be estimated. To calculate the cumulative RID, the infant daily dose (IDD) is first determined by multiplying the drug concentration measured in breast milk by the daily volume of milk consumed [9]. Assuming normal lactation and exclusive breastfeeding, the intake of 150 mL per kg per day defined by the WHO was used [10]. Dividing the IDD by the maternal dose expressed as a percentage gives the RID (%) for that day. Summing the daily RIDs of our case provides the cumulative RID. The formulas used for calculation of the IDD, RID, and cumulative RID are displayed in Supplementary Material S3.

## Results

### Drug level measurements

**Fig. 1:**
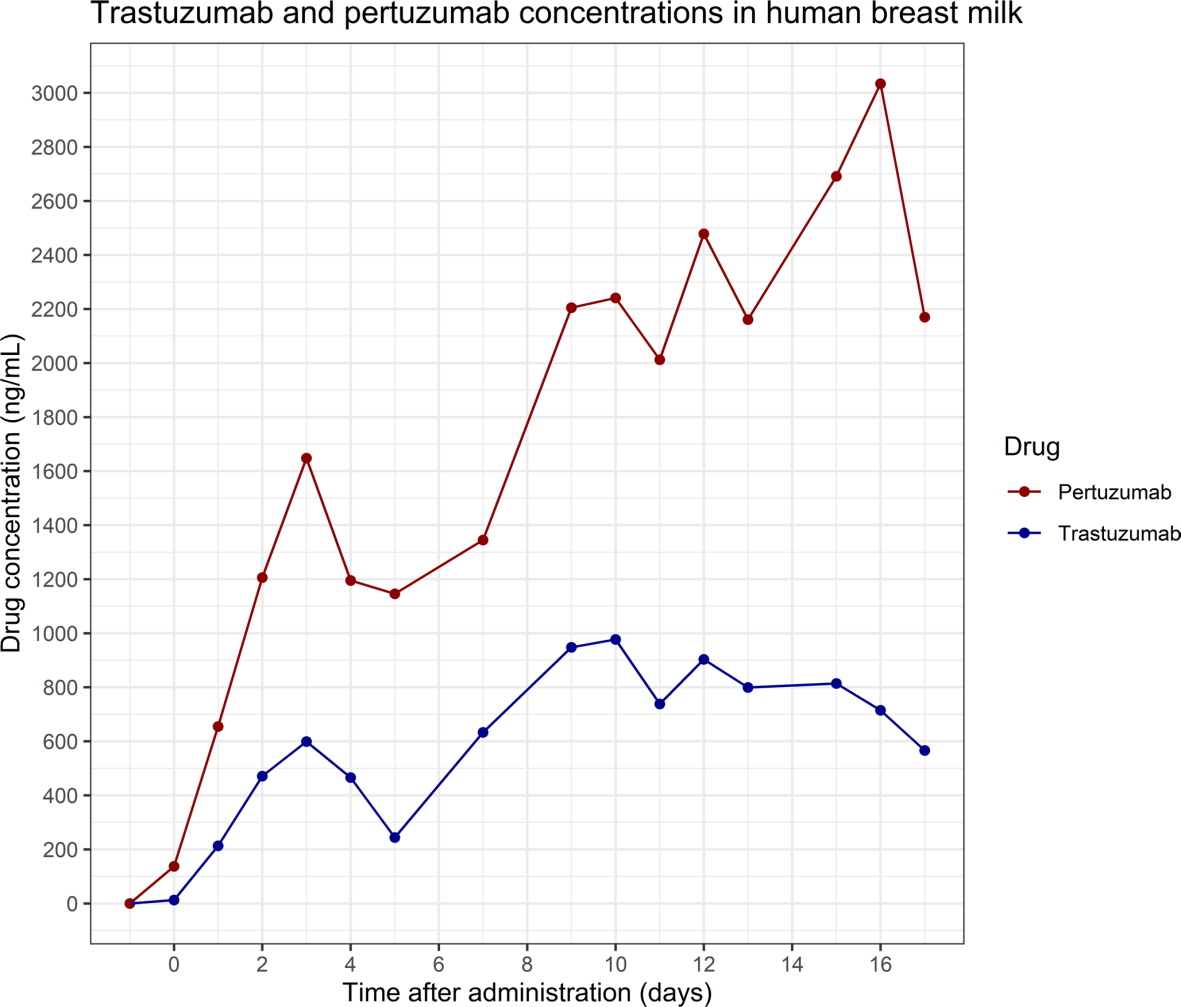
Trastuzumab and pertuzumab concentrations in human breast milk measured across 17 days after IV administration of trastuzumab 600 mg and pertuzumab 1200 mg (t = 0 is day of administration) using two partially validated ELISA methods. Breast milk samples on days 6, 8, and 14 were not collected

Breast milk was collected one day before the start of the first treatment cycle with IV trastuzumab and pertuzumab. Thereafter, breast milk was collected over a 17-day period during the first cycle, until milk production ceased. Daily milk production ranged from five to ten mL, most likely because tamoxifen suppressed lactation. All samples obtained on a given day were pooled into a single collection bag. Breast milk samples were not obtained on days 6, 8, and 14 due to absent milk production. In total, 15 samples were available for quantification of trastuzumab and pertuzumab. Figure 1 shows the trastuzumab and pertuzumab concentration curves over the 17-day time period. Trastuzumab concentrations ranged between 12.7 and 977 ng/mL, whereas concentrations for pertuzumab ranged between 137 and 3034 ng/mL.

### Infant drug exposure

The IDD, RID, and cumulative RID were calculated for both drugs. After the administration of 600 mg trastuzumab, the daily RIDs ranged from 0.02% at the day of administration to a maximum of 1.8% at day 10, resulting in a cumulative RID of 16.8%. Following administration of 1200 mg pertuzumab, the RIDs ranged between 0.1% on the day of administration to 2.8% at day 16, resulting in a cumulative RID of 24.3%. Since sampling was not performed on all days within the first 17 days of treatment, the cumulative RIDs of both drugs were likely underestimated.

## Discussion

Trastuzumab and pertuzumab concentrations in breast milk following IV administration of trastuzumab 600 mg and pertuzumab 1200 mg showed substantial transfer of these monoclonal antibodies into breast milk. For measurement of pertuzumab in human milk, we successfully developed an ELISA. This ELISA and the commercial ELISA we used for trastuzumab were partially validated according to ICH M10 Guideline on Bioanalytical Validation and Study Sample Analysis. Trastuzumab showed a three-peak pharmacokinetic (PK) pattern with a clear plateau after day 9 and a decline from day 15 onward. Pertuzumab showed a four-peak increasing PK pattern, with peak concentrations on day 16 followed by signs of plateau formation. Overall, pertuzumab concentrations exceeded those of trastuzumab by approximately twofold, which correlates with the twofold higher administered dose. This multi-peak pharmacokinetic profile was also described for nivolumab, an IgG_4_, in breast milk [5]. For other IgG_1_ therapeutic antibodies, maximal breast milk concentrations were often reached within 48 h but could also last until 14 days [11].

Exposure parameters were calculated from all breast milk measurements, with the cumulative RID representing the infant exposure across the 17-day sampling period. The applicability of the RID for monoclonal antibodies remains debated, as this value assumes daily maternal dosing rather than intermittent administration as with trastuzumab and pertuzumab [12]. This approach does not account for drug clearance and may, therefore, overestimate the RID. Conversely, incomplete sampling within the 17-day sampling period may have resulted in underestimation of the cumulative RID, as exposure on unsampled days was not captured. Pharmacokinetic modelling could be applied to estimate more accurate RID values and a full-cycle cumulative RID, including unsampled days. Nonetheless, given the long half-lives of these antibodies and the sampling window occurring within one half-life, the calculated RID and cumulative RID values are considered informative. For small non-oncological drugs, an RID safety threshold of 10% has been set to ensure safe breastfeeding while undergoing treatment [13]. The cumulative RIDs of both drugs exceeded this threshold with cumulative RIDs of 16.8% for trastuzumab and 24.3% for pertuzumab. This differs from small-molecule chemotherapeutic agents with shorter half-lives, such as cyclophosphamide and paclitaxel, for which intermittent breastfeeding may be considered to reduce the RID to 0.1% or 1.0% [14].

How the presence of trastuzumab and pertuzumab in breast milk translates into systemic exposure and potential harmful effects in breastfed infants remains uncertain. Pharmacological aspects such as oral availability and metabolism in the breastfed infant play an important role. Combining data from two other IgG_1_-type monoclonal antibodies, could suggest potential uptake and systemic exposure to trastuzumab and pertuzumab. A study on in vivo digestion of palivizumab-fortified human milk showed a 57% reduction of concentration in intestinal samples compared to the concentration in the administered feed [15], indicating substantial proteolysis of monoclonal antibodies after ingestion. Evidence on subsequent absorption of available monoclonal antibodies in the gastrointestinal tract is limited. FcRn-mediated IgG uptake may be possible as FcRn expression has been observed in fetal, child, adolescent, and adult intestinal biopsies [16, 17]. Pharmacokinetic data showed systemic infliximab concentrations after an exclusively breastfed infant with breast milk containing infliximab [6]. Taken together, these findings indicate that potential uptake and systemic exposure to trastuzumab and pertuzumab in breastfed infants cannot be excluded. The extent of uptake and exposure, however, may be lower than the calculated cumulative RID values indicate due to pharmacological processes. Accordingly, breastfeeding during treatment with these agents is not recommended.

## Supplementary Information

Below is the link to the electronic supplementary material.

Supplementary Material 1

## Data Availability

The authors confirm that the data supporting the findings of this study are available upon request.
