# Supplementary material for: Transfer of trastuzumab and pertuzumab into human breast milk: a case report
Source: Cancer Chemother Pharmacol. 2026 May 20;96(1):53. doi: 10.1007/s00280-026-04901-0 (PMC13190791; doi:10.1007/s00280-026-04901-0)
Supplement: Supplementary file 1 — Supplementary Material 1 [file 280_2026_4901_MOESM1_ESM.docx]

## Supplementary Informations

**S1. Measurement of trastuzumab in human breast milk**

Method development
An anti-trastuzumab sandwich ELISA (Trastuzumab (Herceptin®) BioAssay^TM^ ELISA Kit by US Biologicals) was partially validated for its suitability to quantify trastuzumab in human breast milk. Because the assay was originally developed for serum, the use of human breast milk as matrix was validated. In addition, since trastuzumab concentrations in breast milk were expected to be lower than those normally measured in serum, several modifications were explored to improve sensitivity of the assay. Given that pertuzumab was also present in the patient samples, its potential interference was examined. The following adjustments and evaluations were performed. First, samples were diluted 1:10 (v/v) in the kit’s sample buffer rather than the recommended 1:50 (v/v) dilution by the manufacturer, resulting in a final matrix of 10% human breast milk in sample buffer. Calibration standards (2–200 ng/mL) were prepared in the same 10% (v/v) breast milk matrix. To assess potential matrix interference, the manufacturer’s standard-curve samples were diluted only in sample buffer and then quantified against the 10% (v/v) breast milk calibration curve. Accuracies fell within ±11.2% of the nominal values, confirming the suitability of the modified assay conditions for breast milk analysis. Second, background signal from the 10% (v/v) human breast milk matrix was substantially reduced by introducing an additional two-hour blocking step at 2-8˚C using 300 µL phosphate buffered saline with 0.1% (v/v) Tween-20 and 10% (v/v) Ficoll (PBSTF10%) prior to sample application. Third, an alternative detection antibody, mouse anti-human IgG_1_ Fc antibody-HRP conjugate from ThermoFisher (Landsmeer, The Netherlands), was evaluated. However, compared with the kit-supplied antibody, it produced a higher background signal and resulted in a 20% loss of sensitivity. Finally, potential interference from pertuzumab was tested at concentrations of 5, 20, and 100 ng/mL. Signals were comparable to blank controls, confirming that pertuzumab does not interfere with trastuzumab quantification under these assay conditions.

Ultimately, we were able to quantify trastuzumab in 10% (v/v) human breast milk in sample buffer over a linear range of 1-20.0 ng/mL using five calibration standards (1, 2, 5, 10, and 20 ng/mL) and three quality control (QC) levels (2, 5, and 15 ng/mL). Stability of trastuzumab in human breast milk was assessed after 24 hours of storage on ice and after five weeks of storage at -20˚C, covering the time after collection and measurement. Both stability samples were analyzed in triplicate and showed deviations of -2.62% and -4.15%, respectively from the nominal concentration (15 ng/mL in 10% human breast milk matrix). Based on these results, the stored patient breast milk samples were analyzed using dilution factors of 10, 100, 200, and 300, depending on sampling time, while maintaining a final matrix of 10% (v/v) human breast milk in sample buffer.

Final sample work-up protocol
Two hours prior to sample loading, wells were blocked with 300 µL PBSTF10%. Calibration curve samples (1, 2, 5, 10, and 20 ng/mL) and QC samples (2, 5, and 15 ng/mL) were prepared in 10% (v/v) blank human breast milk in sample buffer. Patient samples were diluted 10-, 100-, 200-, and 300-fold depending on the sampling time. Subsequently, 100 µL of each sample was applied to the plate, and the rest of the manufacturer protocol was followed to determine the trastuzumab concentrations in breast milk.

**S2. Measurement of pertuzumab in human breast milk**

Method development
Two commercial kits were initially evaluated for quantification of pertuzumab concentrations in human breast milk. First, a KTR 755 humanized anti-HER2/neu (Herceptin/trastuzumab) by Epitope Diagnostics was selected to measure the total concentration of both anti-HER2 antibodies, trastuzumab and pertuzumab, from which the pertuzumab concentration could be derived by subtraction of the already measured trastuzumab concentration. However, during assay evaluation, we observed a 3,000-fold lower signal for pertuzumab compared with trastuzumab, as well as a substantial background signal when using 10% (v/v) human breast milk in the sample buffer matrix. Consequently, this kit was found unsuitable for the determination of both drugs. A second assay, an anti-pertuzumab sandwich ELISA (Pertuzumab ELISA kit) by Raybiotech, was then tested. Although being selective for pertuzumab, this kit was also not suitable for our application since the presence of human breast milk in the sample matrix led to very low pertuzumab signals. Reducing the breast milk concentration to 1% (v/v) and more extensive centrifugation did not improve performance. The combination of the breast milk matrix and the plate material may have contributed to the assay failure.

Since neither commercial kit was suitable, we proceeded to develop an in-house ELISA method. Our first attempt was a sandwich ELISA using a human anti-pertuzumab antibody (clone AbD37070) with a HuCAL Fab-monovalent isotype by BioRad. While selective for pertuzumab, calibration standards in PBSTF10% produced lower than expected signals, and the addition of human breast milk resulted in almost complete signal loss. Using centrifuged or non-fat breast milk did not improve the assay. We, therefore, developed another ELISA method using a full mouse anti-pertuzumab antibody (1A3, A0215040) by GenScript®, using the experience and protocol from an earlier published ELISA method for the quantification of ipilimumab in human breast milk by Pluim *et al.* (2024). Similar to the trastuzumab assay, we optimized sensitivity by evaluating different blocking conditions, and we evaluated potential interference by trastuzumab. Pre-sample loading blocking with PBSTF10% alone or in combination with 2% (v/v) or 4% (v/v) Blotto non-fat dry milk (SC-2325) by Santa Cruz Biotechnology was tested. The addition of 2% or 4% Blotto non-fat dry milk increased the signal-to-noise ratio by twofold, therefore, 2% Blotto non-fat dry milk in PBSTF10% was selected for all subsequent experiments.
Using the optimizations, we were able to develop an anti-pertuzumab sandwich ELISA that quantifies pertuzumab in 10% human breast milk in PBSTF10% in a range of 1-50 ng/mL using six calibration standards (1, 2, 5, 10, 20, and 50 ng/mL), and three QC levels (2, 10, and 50 ng/mL). This range was linear, accurate (intra-run bias ±13.5%, inter-run bias ±3.7%), and precise (intra-run precision ±10.6%, inter-run precision ±10.6%). Interference of trastuzumab was assessed at 2, 10, and 50 ng/mL and resulted in an absence of signal, confirming specificity for pertuzumab. Stability testing indicated that pertuzumab remained stable in breast milk for 24 hours on ice and for at least 8 months at -20˚C, reflected by the deviation from nominal values by only ±0.33% and ±7.94%, respectively, at 10 and 50 ng/mL pertuzumab. Based on these results and depending on sampling time, patient breast milk samples were analyzed after 10-, 100-, 200-, and 300-fold dilution with blank 10% (v/v) human breast milk in PBSTF10%.

Final sample work-up protocol
A transparent Nunc MaxiSorp® plate was coated overnight at 4˚C with 50 µL mouse anti-pertuzumab IgG (1.84 µg/mL). Wells were then blocked for 1-2 hours at 4˚C with 250 µL of 2% (v/v) Blotto non-fat dry milk in PBSTF10%. After blocking, the plate was washed 4 times with 300 µL PBSTF1%, after which 50 µL of the calibration standards, QC samples, and patient breast milk samples were added. Samples were incubated for 2 hours at room temperature with 100 rpm shaking. Following another four washes with PBSTF1%, 50 µL of a 1:500 dilution of mouse anti-IgG_1_-HRP conjugate from ThermoFisher (Landsmeer, The Netherlands) in PBS was added and subsequently incubated for 1 hour at room temperature with 100 rpm shaking. After a final washing step with 4 times 300 µL PBSTF1%, 50 µL of Ultra-TMB from ThermoFisher (Landsmeer, The Netherlands) was added and incubated for 10 minutes at 100 rpm shaking. The reaction was stopped using 100 µL 2M H_2_SO_4_, and absorbance was measured at 450 nm with background correction at 595 nm using a Tecan Infinite 200 Pro plate reader at 1 second read time per well.

**S3. Formulas used to calculate child exposure to trastuzumab and pertuzumab through breastfeeding**

For both drugs, the following formulas were used to calculate the infant daily dose (IDD), relative infant dose (RID), and cumulative RID.

$Infant daily dose \left( IDD \right) (\frac{\frac{ng}{kg}}{day})= Concentration measured in breast milk \left( \frac{ng}{mL} \right)x volume breast milk consumed daily (\frac{\frac{mL}{kg}}{day})$

$Relative infant dose \left( RID \right) \left( \% \right)$ $=\frac{infant daily dose (\frac{\frac{ng}{kg}}{day})}{maternal daily dose (\frac{\frac{ng}{kg}}{day})} x 100\%$

$Cumulative RID \left( \% \right)= \sum_{i=1 cylce}^{n=17 days} {RID}_{i}(\%)$
